# Efficacy and safety of oral zinc sulfate in the prevention of chemotherapy-induced oral mucositis: Protocol for a meta-analysis of randomized controlled trials

**DOI:** 10.1097/MD.0000000000010839

**Published:** 2018-05-25

**Authors:** Xu Tian, Wei-Qing Chen, Xiao-Ling Liu, Yuan-Ping Pi, Hui Chen

**Affiliations:** a Chongqing Key Laboratory of Translational Research for Cancer Metastasis and Individualized Treatment; b Key Laboratory for Biorheological Science and Technology of Ministry of Education (Chongqing University), Chongqing University Cancer Hospital & Chongqing Cancer Institute & Chongqing Cancer Hospital, Chongqing, China.

**Keywords:** chemotherapy, meta-analysis, oral mucositis, systematic review, zinc sulfate

## Abstract

**Background:**

Oral mucositis has been an extremely serious complication resulted from cytotoxic effects of the chemotherapy among cancer patients. Several randomized controlled trials investigated the efficacy of zinc sulfate in prevention of this morbid condition among cancer patients undergoing chemotherapy, however conclusive findings has not yet been generated. This systematic review will assess the efficacy and safety of oral zinc sulfate for chemotherapy-induced oral mucositis.

**Methods:**

We will electronically search all potential citations in PubMed, EMBASE, and Cochrane Central Register of Controlled Trials (CENTRAL) from their inception to April 2018. The randomized controlled trials, which investigated the efficacy of oral zinc sulfate for chemotherapy-induced oral mucositis will be considered. We will assign 2 independent investigators to perform search, screen citations, extract data, and appraise risk of bias. And then, the primary investigator will adopt RevMan 5.3 software to complete all statistical analyses.

**Ethics and dissemination:**

The findings from this systematic review and meta-analysis will be submitted to a peer-reviewed journal for publication. Moreover, we will disseminate all results in any topic-related conference.

**Registration number:**

This protocol has been registered with a number of CRD42018093605.

## Abstract 2

Supplemental Digital Content is available in the text

## 1 Introduction

Oral mucositis (OM) is a morbid condition that appears as a significant result of a series of inflammatory changes in the epithelial and subepithelial cells of the oral mucosa due to the cytotoxic effects of the chemotherapy or radiotherapy.^[1,2]^ Studies reported that 81.3% and 90% of patients with cancer^[3]^ and acute leukemia^[4]^ received the treatment with chemotherapy suffered from oral mucositis. Cancer patients will experience pain, physical limitations, and psychological discomfort after being diagnosed with the oral mucositis.^[4]^ Moreover, this debilitating condition also significantly affect nutritional intake, mouth care and quality of life, as well as increase economic burden.^[4–7]^

Although the incidence of oral mucositis during cancer therapies and corresponding consequences are extremely serious, the prophylaxis and treatment which is targeted to this given condition remains an unsolved problem.^[7,8]^ More importantly, the effects of interventions which have been also used in this regard such as low-level laser therapy and several organic products^[9]^ have not yet been completely confirmed.^[7]^ Evidence suggested that zinc can increase the gastrointestinal epithelial barrier function and consequently decrease cell death and detachment.^[10]^ Several studies have been performed to investigate the efficacy of zinc for chemotherapy-induced oral mucositis.^[7,11–14]^ Of these studies, four^[7,11,12,14]^ supported zinc to reduce the incidence and severity of mucositis in cancer patients undergoing chemotherapy. However, Mansouri et al^[13]^ suggested no benefit when zinc was prescribed for high-dose chemotherapy-induced mucositis. And thus, the efficacy and safety of zinc for chemotherapy-induced oral mucositis remains controversial. We consequently performed this systematic review and meta-analysis to comprehensively assess the effect and safety of zinc sulfate for chemotherapy-induced oral mucositis. We designed this systematic review and meta-analysis on 10 March 2018 and we expected to complete this study by June 30, 2018.

## 2 Methods and design

**Figure 1:**
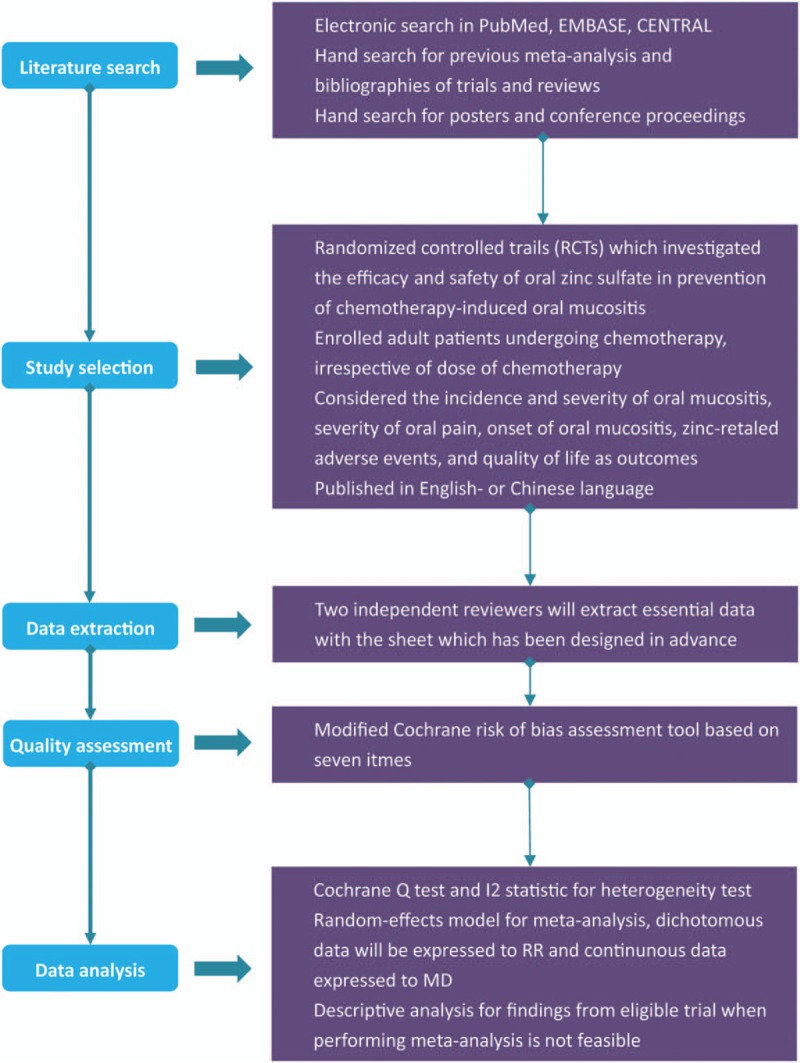
Process of the systematic review and meta-analysis. CENTRAL = Cochrane Central Register of Controlled Trials, RCTs = randomized controlled trials, RR = relative risk, MD = mean difference.

We designed this protocol for a systematic review and meta-analysis according to the framework constructed in preferred reporting items for systematic reviews and meta-analysis protocols (PRISMA-P) 2015: elaboration and explanation.^[15]^ The systematic review and meta-analysis has been registered in the International Prospective Register of Systematic Reviews (PROSPERO) platform with the number of CRD42018093605. We will perform all statistical analyses in accordance with the recommendations proposed by Cochrane Collaboration (CC).^[16]^ The process of the whole study was displayed in Figure 1.

### 2.1 Selection criteria

In our meta-analysis, a study will be included if the following inclusion criteria are met: patients: all patients which are aged between 15 years and older undergoing chemotherapy, irrespective of dose of chemotherapy; intervention: the patients in study group were instructed to oral zinc sulfate, and patients in control group took placebo capsules or other active drugs that were similar in shape, taste, and color to the zinc sulfate capsules; outcomes: incidence and severity of oral mucositis were defined as the primary outcome, and the secondary outcomes included severity of oral pain, onset of oral mucositis, zinc-related adverse events (AEs) and quality of life (QoL); study design: only randomized controlled trials (RCTs) will be included, however an abstract with sufficient data will also be considered; and language: only full text published in the English or Chinese language will be considered, because translators well versed in other languages are not included.

A study will be excluded if it meets at least one of the following criteria: essential information cannot be extracted; duplication with poor methodology and insufficient data; non-original research types, such as review, editorial, letter to the editor, or comments; a study investigating bowel preparation regimen in special patients, such as the patients with a previous oral mucositis or other mucositis; and trials including patients who are receiving radiotherapy or chemoradiotherapy will be excluded.

### 2.2 Definition of outcomes

In our systemic review and meta-analysis, incidence of oral mucositis was defined as the value of number of experienced the oral mucositis irrespective of grade divided by the total number of cancer patients completed the whole study, and the severity of oral mucositis was graded using the validated scale including World Health Organization (WHO) criteria^[17]^ and Spijkervet scale.^[18]^ The severity of oral pain was evaluated based on a visual analog scale (VAS).^[7]^ Onset of oral mucositis was defined as the time of definitively diagnosed oral mucositis. Zinc-related adverse events referred to any adverse event resulted from oral zinc sulfate. The quality of life was assessed by using validated scales such as European Organization for Research and Treatment of Cancer (EORTC) LQ-OES18.^[11]^

### 2.3 Identification of citations

**Figure 2:**
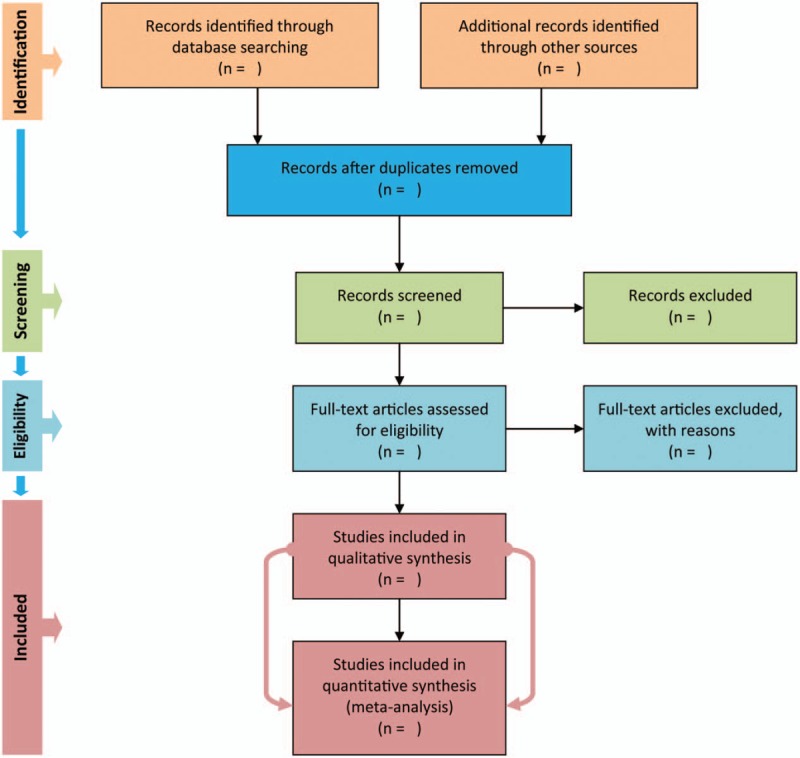
PRISMA flow chart. PRISMA = preferred reporting items for systematic reviews and meta-analysis. This process will be completed by two independent reviewers according to the inclusion and exclusion criteria.

We will electronically search PubMed, Cochrane Central Register of Controlled Trials (CENTRAL), and EMBASE in order to capture all potential records investigating the efficacy and safety of oral zinc sulfate in the prevention of chemotherapy-induced oral mucositis from their inception to April 2018. Words such as ‘zinc’, ‘oral mucositis’ and ‘random’ will be used to construct search algorithms in accordance with the requests of targeted databases, and all possible search algorithms have been documented in the supplemental search strings (Supplemental Digital Content 1). After the electronic searches, we will also hand check the reference lists of all eligible studies and topic related reviews and electronically retrieve the Clinicaltrial.gov for the purpose of covering all potential eligible studies. However, only studies published in English and Chinese will be considered in our systematic review and meta-analysis. The PRISMA flow chat^[19]^ will be used to depict the process of searching and screening citations (Fig. 2).

### 2.4 Data extraction

We have designed a standard data extraction form, used in our previous systematic reviews and meta-analyses (see Supplemental Digital Content 2: supplemental data extraction table). All captured citations will be imported into EndNote literature management software V.X7. We will then assign 2 reviewers to abstract the basic information and data for the specific outcomes from the eligible studies, such as first author, publication year, age of participants, sample size, details of interventions, and outcomes of interest using this standard data extraction form. We will contact the corresponding author if sufficient data of an eligible study cannot be abstracted from the full text.

### 2.5 Quality assessment of individual study

We will assign two independent reviewers to appraise the risk of bias from seven domains, including randomization sequence generation, allocation concealment, blinding of participants, blinding of study personnel, blinding of outcome assessors, incomplete outcome data, selective reporting and other bias with the Cochrane risk of bias assessment tool.^[16,20]^ A study will be assigned a risk level of ‘high risk of bias’, ‘unclear risk of bias’ or ‘low risk of bias’ according to the match level between the actual information and the evaluation criteria.^[16]^

### 2.6 Statistical analysis

For calculating the pooled effects, the dichotomous data will be expressed as relative risk (RR) with 95% confidence intervals (CIs), and continuous data will be extracted as mean difference (MD) with 95% CIs. And then, we will perform traditional pair wise meta-analysis based on the random-effect model, which incorporates within and between studies heterogeneity, to estimate the summarized RR and 95% CIs.^[21]^ The χ^2^ method will be adopted to test the heterogeneity^[22]^ and the *I*^*2*^ statistic will be used to estimate the proportion of the overall variation that is attributable to between study heterogeneity.^[23]^ A value for the *I*^*2*^ statistic >50% indicates substantial heterogeneity.^[23]^ The studies with more than 2 comparison groups will be quantitatively incorporated into the pair wise meta-analysis according to the specific comparison.^[16]^ All analyses will be conducted using the RevMan 5.3 (Copenhagen, Denmark: the Nordic Cochrane Center, the Cochrane Collaboration, 2013) and Stata 12 (StataCorp, TX).

### 2.7 Subgroup and sensitivity analyses

In the case of possible important heterogeneity, we will explore the possible sources using subgroup and meta-regression analyses. Subgroup analyses are planned for dose of chemotherapy and control regimes. Sensitivity analyses are planned for prevention of chemotherapy-induced oral mucositis by analyzing only studies considered at low risk of bias.

### 2.8 Publication bias

For single outcome, We will draw the funnel plot to identify publication bias if the number of studies analyzed is more than 10.^[24]^ Moreover, we will also perform the Egger linear regression test to quantitatively detect the symmetric or a symmetric of funnel plot.^[25]^

## 3 Discussion

Cancer has been a major public health problem worldwide and is the first leading cause of death in the mainland China. Chemotherapy, radiotherapy or both is still the important option in the treatment of cancer, however cytotoxic effects of these methods are also associated with short and long term side effects,^[26]^ for example, chemo/radiotherapy-induced oral mucositis.^[11]^ Evidences suggested that more than 40% of cancer patients undergoing chemo-radiotherapy suffered from oral mucositis.^[27]^ However, it is noted that occurrence of oral mucositis among cancer patients is mainly depending on the cytotoxic regimen and patient-associated variables.^[28]^ For example, the prevalence is between 10% and 100% in patients receiving systemic anticancer chemotherapy or local irradiation for tumors in the head and neck area.^[2]^ Whereas, other studies revealed that 81.3% and 90% of patients with cancer^[3]^ and acute leukemia^[4]^ under chemotherapy suffered from mucositis. As a debilitating condition, cancer patients will experience pain, physical limitations, and psychological discomfort when oral mucositis was definitively diagnosed.^[4]^ Moreover, cancer patients with chemo/radiotherapy-induced oral mucositis was also associated with decreased nutritional intake, mouth care and quality of life.^[5]^ Certainly, oral mucositis also increased medical expenditure of cancer patients because this condition will cause prolonged length of hospital stay and poor quality of life. For example, a study on malignant hematologic patients published in 2014 suggested that oral mucositis was associated with the 21.9% of hospitalization episodes,^[4]^ and another study found that anti-cancer treatment will be disrupted when severe inflammation causes marked pain.^[7]^

Many methods have been developed to prevent and treat chemo/radiotherapy-induced oral mucositis because this condition significantly affects prognosis of cancer patients, however the prophylaxis and treatment of oral mucositis during cancer therapies is still an unsolved problem.^[7,8]^ More importantly, the effects of these all interventions have not been completely confirmed.^[7]^ Published evidences demonstrated that zinc performs as an organelle stabilizer and a stabilizer of the structure of DNA, RNA, and ribosome.^[29]^ Moreover, some studies also indicated that zinc is a significant cofactor for DNA synthesis, an important factor for wound healing, and a necessary trace component for improving the immune system.^[30]^ And thus, researchers investigated the efficacy of zinc in preventing and treating chemo/radiotherapy-induced oral mucositis among cancer patients undergoing anticancer treatment, and most studies found promising results.^[31,32]^ It is essential to separately investigate the efficacy of zinc in prevention of chemotherapy-induced oral mucositis due to the differences between radiotherapy and chemotherapy cannot be ignored.^[33]^ To date, five studies^[7,11–14]^ explored the efficacy of zinc for chemotherapy-induced oral mucositis, of which four^[7,11,12,14]^ found that zinc reduced the incidence and severity of mucositis in cancer patients undergoing chemotherapy; however, Mansouri et al^[13]^ found that zinc did not have any clinical benefits in prevention or reduction of severity, and duration of high-dose chemotherapy-induced mucositis. And thus, it remains a controversial on the efficacy of zinc for the treatment of chemotherapy-induced oral mucositis. So we consequently performed this systematic review and meta-analysis to comprehensively assess the effect and safety of zinc sulfate for chemotherapy-induced oral mucositis.

This systematic review and meta-analysis will be one of the first to investigate the comparative efficacy and safety of oral zinc sulfate in prevention of chemotherapy-induced oral mucositis. The results of the systematic review and meta-analysis will influence evidence based decision making for chemotherapy-induced oral mucositis prevention as it will be fundamental in providing reliable recommendations for chemotherapy-induced oral mucositis prevention.

### 3.1 Ethics and dissemination

Ethics approval and patient written informed consent will not be required because all analyses in the present study will be performed based on data from published studies. We will submit our systematic review and meta-analysis to a peer reviewed scientific journal for publication.

## Author contributions

XT and WQC conceived the study, developed the study criteria. XT and XLL searched the literature. YPP and HC analyzed the data. XT, WQC, HH, and JW wrote the protocol. HH and JW conducted the preliminary search. XT and HC extracted data. WQC and YPP revised the article. All authors have read, and approved the final article.

**Conceptualization:** Xu Tian, Wei-Qing Chen, Yuan-Ping Pi.

**Data curation:** Xiao-Ling Liu, Hui Chen.

**Formal analysis:** Xu Tian, Hui Chen.

**Investigation:** Wei-Qing Chen, Xiao-Ling Liu, Yuan-Ping Pi, Hui Chen.

**Methodology:** Xu Tian, Wei-Qing Chen, Yuan-Ping Pi.

**Software:** Xu Tian.

**Supervision:** Wei-Qing Chen.

**Writing – original draft:** Xu Tian, Wei-Qing Chen.

**Writing – review & editing:** Xu Tian, Wei-Qing Chen.

## Supplementary Material

Supplemental Digital Content
