# Supplementary material for: Efficacy and safety of oral zinc sulfate in the prevention of chemotherapy-induced oral mucositis: Protocol for a meta-analysis of randomized controlled trials
Source: Medicine (Baltimore). 2018 May 25;97(21):e10839. doi: 10.1097/MD.0000000000010839 (PMC6392683; doi:10.1097/MD.0000000000010839)
Supplement: Supplemental Digital Content [file medi-97-e10839-s001.pdf]

# Search strings for targeted databases

## *PubMed search algorithm*

| Search | Query |
|---|---|
| #8 | Search ((("Zinc Sulfate"[Mesh]) OR ((((((zinc Sulfate[Title/Abstract]) OR solvazinc[Title/Abstract]) OR verazinc[Title/Abstract]) OR zinc sulphate[Title/Abstract]) OR zincomed[Title/Abstract]) OR zincteral[Title/Abstract]))) AND (((("Mucositis"[Mesh]) OR "Stomatitis"[Mesh])) OR (((((((((Mucositides[Title/Abstract]) OR mucosa irritation[Title/Abstract]) OR mucositis[Title/Abstract]) OR mucosa inflammation[Title/Abstract]) OR Stomatitis[Title/Abstract]) OR Stomatitides[Title/Abstract]) OR Oral Mucositis[Title/Abstract]) OR Oral Mucositides[Title/Abstract]) OR Oromucositis[Title/Abstract]) OR Oromucositides[Title/Abstract]))) AND random* |
| #7 | Search random* |
| #6 | Search ((("Mucositis"[Mesh]) OR "Stomatitis"[Mesh])) OR (((((((((Mucositides[Title/Abstract]) OR mucosa irritation[Title/Abstract]) OR mucositis[Title/Abstract]) OR mucosa inflammation[Title/Abstract]) OR Stomatitis[Title/Abstract]) OR Stomatitides[Title/Abstract]) OR Oral Mucositis[Title/Abstract]) OR Oral Mucositides[Title/Abstract]) OR Oromucositis[Title/Abstract]) OR Oromucositides[Title/Abstract]) |
| #5 | Search (((((((((Mucositides[Title/Abstract]) OR mucosa irritation[Title/Abstract]) OR mucositis[Title/Abstract]) OR mucosa inflammation[Title/Abstract]) OR Stomatitis[Title/Abstract]) OR Stomatitides[Title/Abstract]) OR Oral Mucositis[Title/Abstract]) OR Oral Mucositides[Title/Abstract]) OR Oromucositis[Title/Abstract]) OR Oromucositides[Title/Abstract] |
| #4 | Search ("Mucositis"[Mesh]) OR "Stomatitis"[Mesh] |
| #3 | Search ("Zinc Sulfate"[Mesh]) OR ((((((zinc Sulfate[Title/Abstract]) OR solvazinc[Title/Abstract]) OR verazinc[Title/Abstract]) OR zinc sulphate[Title/Abstract]) OR zincomed[Title/Abstract]) OR zincteral[Title/Abstract]) |
| #2 | Search (((((zinc Sulfate[Title/Abstract]) OR solvazinc[Title/Abstract]) OR verazinc[Title/Abstract]) OR zinc sulphate[Title/Abstract]) OR zincomed[Title/Abstract]) OR zincteral[Title/Abstract] |
| #1 | Search "Zinc Sulfate"[Mesh] |

***EMBASE search algorithm***

| No. | Query |
|---|---|
| #8 | #3 AND #6 AND #7 |
| #7 | random* |
| #6 | #4 OR #5 |
| #5 | 'mucositis':ti,ab,kw OR 'mucositides':ti,ab,kw OR 'mucosa irritation':ti,ab,kw OR 'mucosa inflammation':ti,ab,kw OR 'oral mucositis':ti,ab,kw OR 'stomatitides':ti,ab,kw OR 'stomatitis':ti,ab,kw OR 'oral mucositides':ti,ab,kw OR 'oromucositis':ti,ab,kw OR 'oromucositides':ti,ab,kw |
| #4 | 'oral mucositis'/exp OR 'mucosa inflammation'/exp OR 'stomatitis'/exp |
| #3 | #1 OR #2 |
| #2 | 'zinc sulfate':ti,ab,kw OR 'solvazinc':ti,ab,kw OR 'verazinc':ti,ab,kw OR 'zinc sulphate':ti,ab,kw OR 'zincomed':ti,ab,kw OR 'zincteral':ti,ab,kw |
| #1 | 'zinc sulfate'/exp |

***CENTRAL search algorithm***

| No. | Query |
|---|---|
| #1 | solvazinc:ti,ab,kw or verazinc:ti,ab,kw or zinc sulphate:ti,ab,kw or zincomed:ti,ab,kw or zincteral:ti,ab,kw in Trials (Word variations have been searched) |
| #2 | MeSH descriptor: [Zinc Sulfate] explode all trees |
| #3 | #1 or #2 |
| #4 | Mucositides:ti,ab,kw or mucosa irritation:ti,ab,kw or mucositis:ti,ab,kw or mucosa inflammation:ti,ab,kw or Stomatitis:ti,ab,kw or stomatitides:ti,ab,kw or Oral Mucositides:ti,ab,kw or oral mucositis:ti,ab,kw or Oromucositis:ti,ab,kw or Oromucositides:ti,ab,kw (Word variations have been searched) |
| #5 | MeSH descriptor: [Mucositis] explode all trees |
| #6 | MeSH descriptor: [Stomatitis] explode all trees |
| #7 | #4 or #5 or #6 |
| #8 | random* (Word variations have been searched) |
| #9 | #3 and #7 and #8 |

Supplemental Digital Content 1: supplemental search strings for all targetd databases.
Supplemental Digital Content 2: supplemental data extraction table.

# Data Extraction Table Designed by Xu Tian

**Summary of Included Trial Characteristics**

| | | **Trial 1** | **Trial 2** | **Trial 3** |
|---|---|---|---|---|
| | | | | |
| Design | | | | |
| Duration of follow-up | | | | |
| Location | | | | |
| | | | | |
| Total number | | | | |
| Age | | | | |
| Sex (male/female) | | | | |
| Baseline characteristics | | | | |
| Assessment of compliance | | | | |
| | | | | |
| Intervention | | | | |
| Control | | | | |
| Other comparator/s | | | | |
| | | | | |
| Primary | | | | |
| Secondary | | | | |
| Analysis method | | | | |
| Notes | | | | |
| | | | | |
| Random sequence generation (selection bias) | Support for judgement | | | |
| | Author's judgement | Unclear risk | Low risk | Unclear risk |
| Allocation concealment (selection bias) | Support for judgement | | | |
| | Author's judgement | Low risk | Low risk | Unclear risk |
| Blinding (performance bias and detection bias) Investigators and Patients | Support for judgement | | | |
| | Author's judgement | Low risk | Low risk | Unclear risk |
| Blinding (performance bias and detection bias) [illegible] | Support for judgement | | | |

| | | | | |
|---|---|---|---|---|
| Outcome assessors | Author's judgement | High risk | High risk | Unclear risk |
| Incomplete outcome data (attrition bias) All outcomes | Support for judgement | | | |
| | Author's judgement | Low risk | Low risk | Low risk |
| Selective reporting (reporting bias) | Support for judgement | | | |
| | Author's judgement | High risk | Low risk | Low risk |
| Other bias | Support for judgement | | | |
| | Author's judgement | Low risk | Low risk | Low risk |
